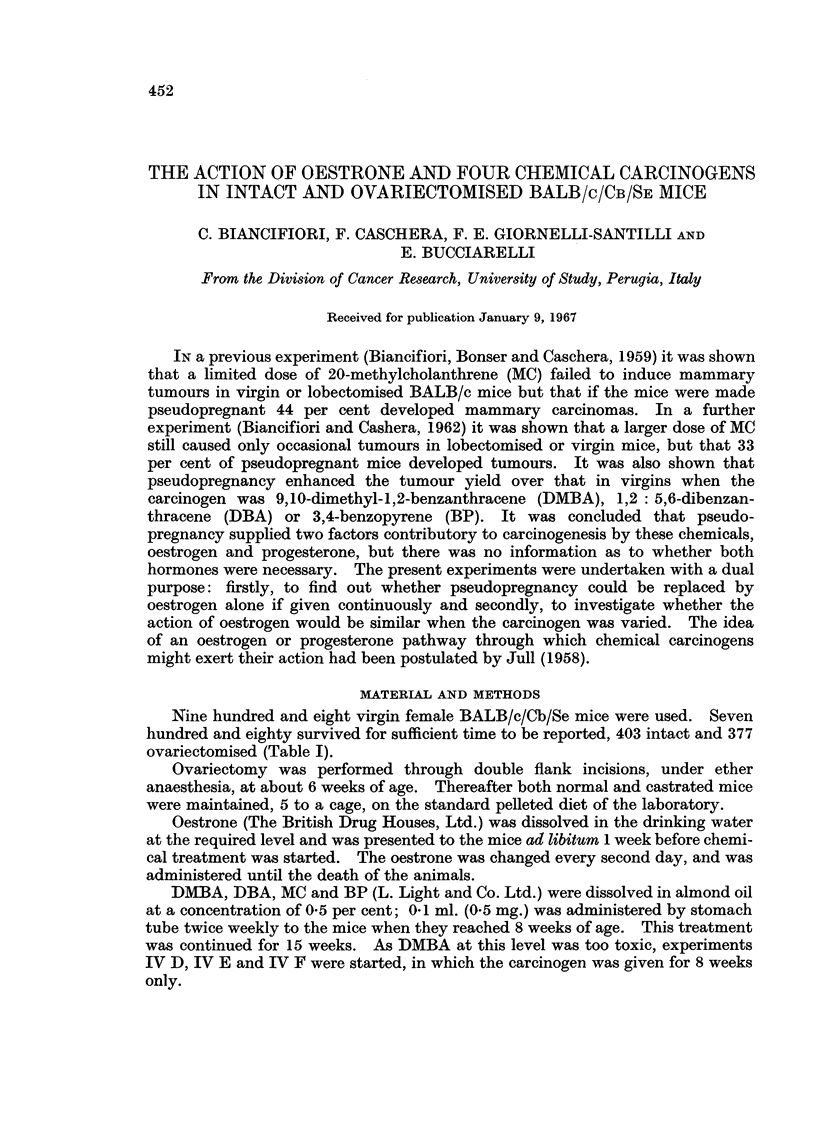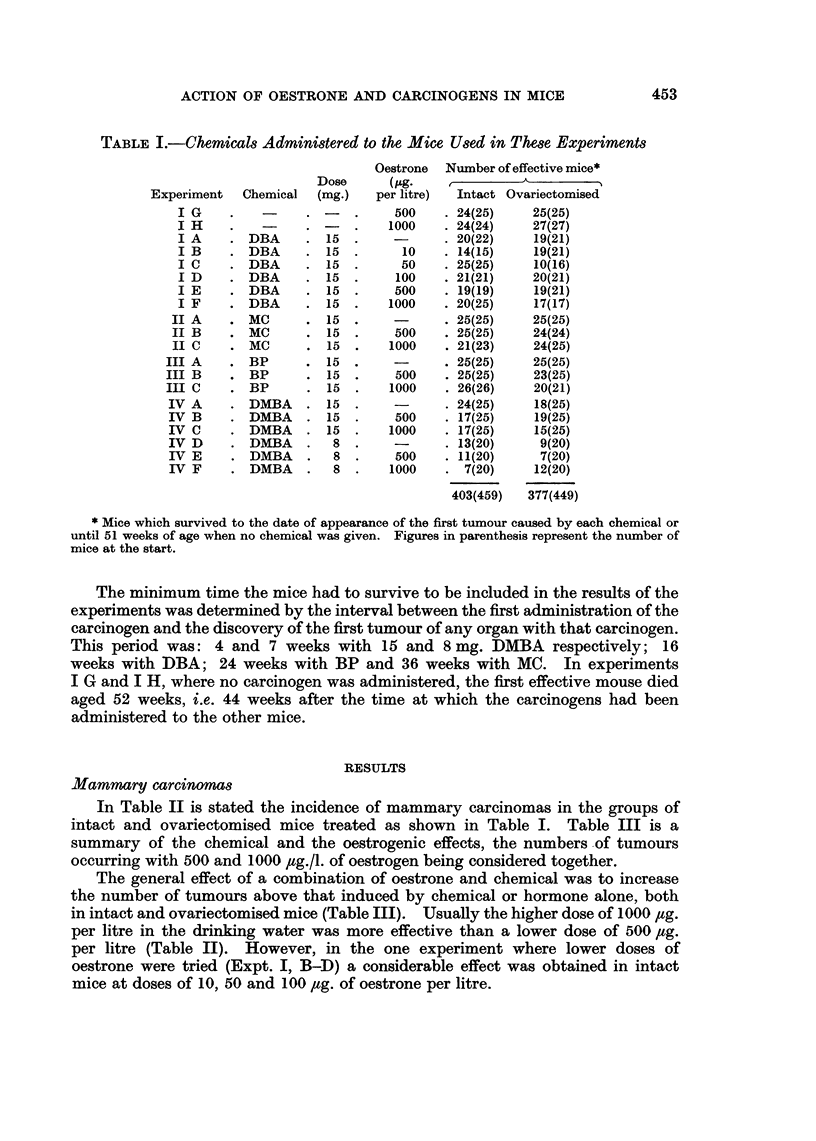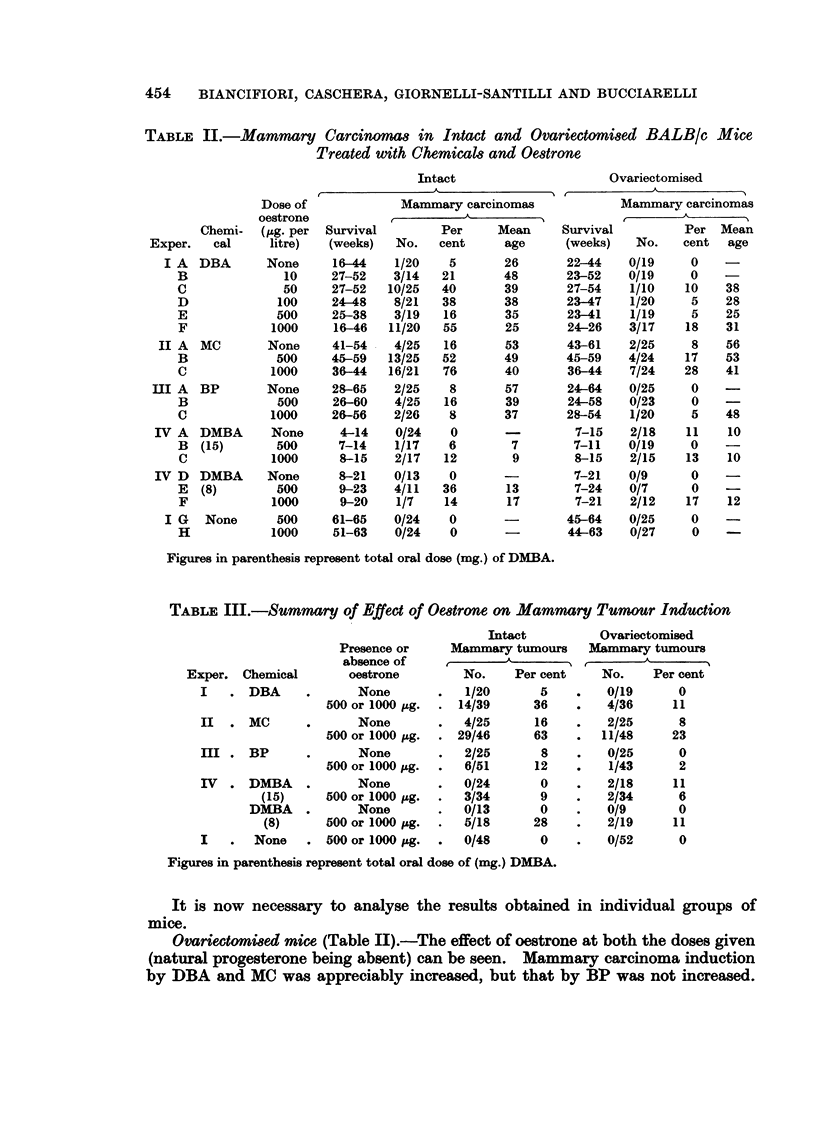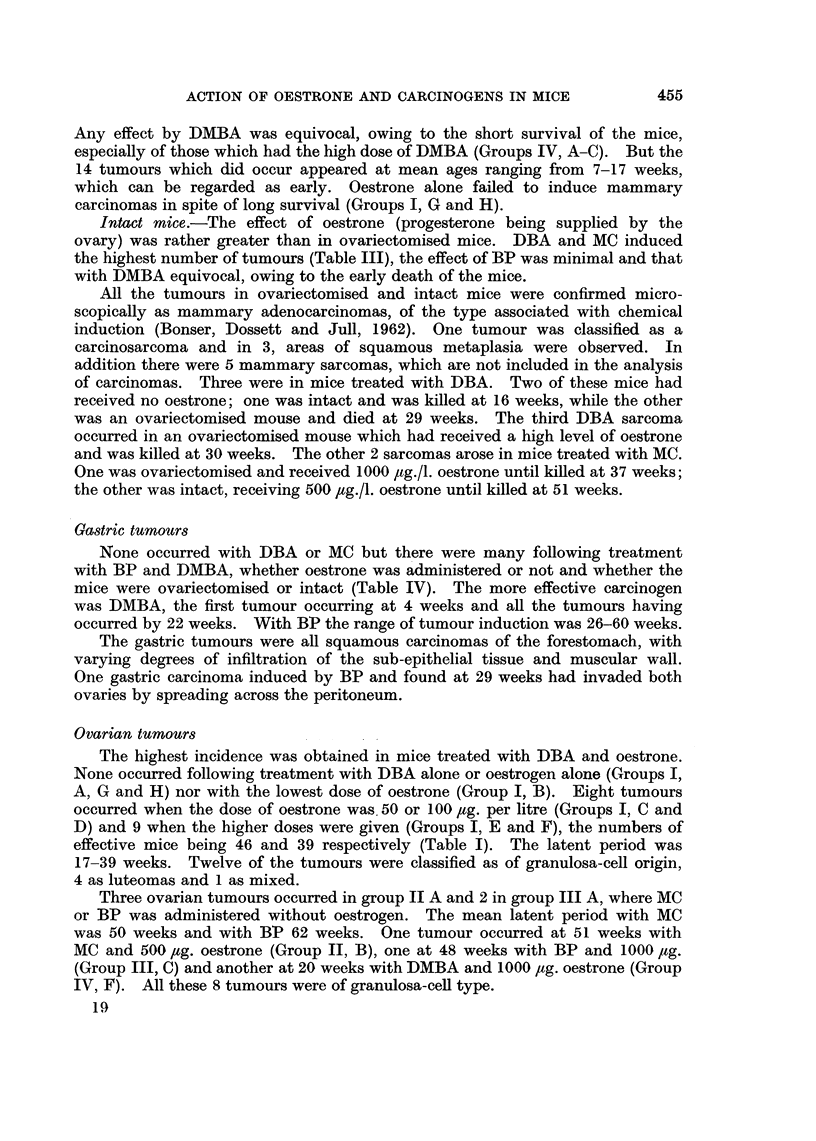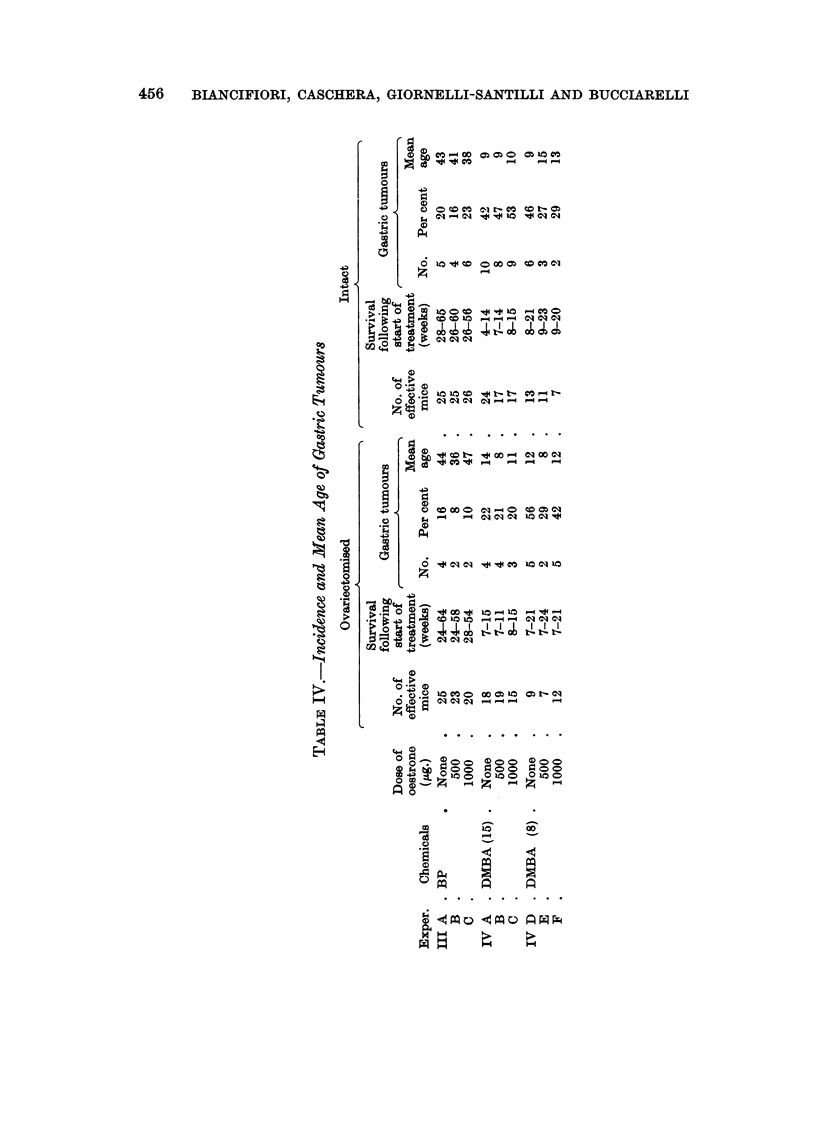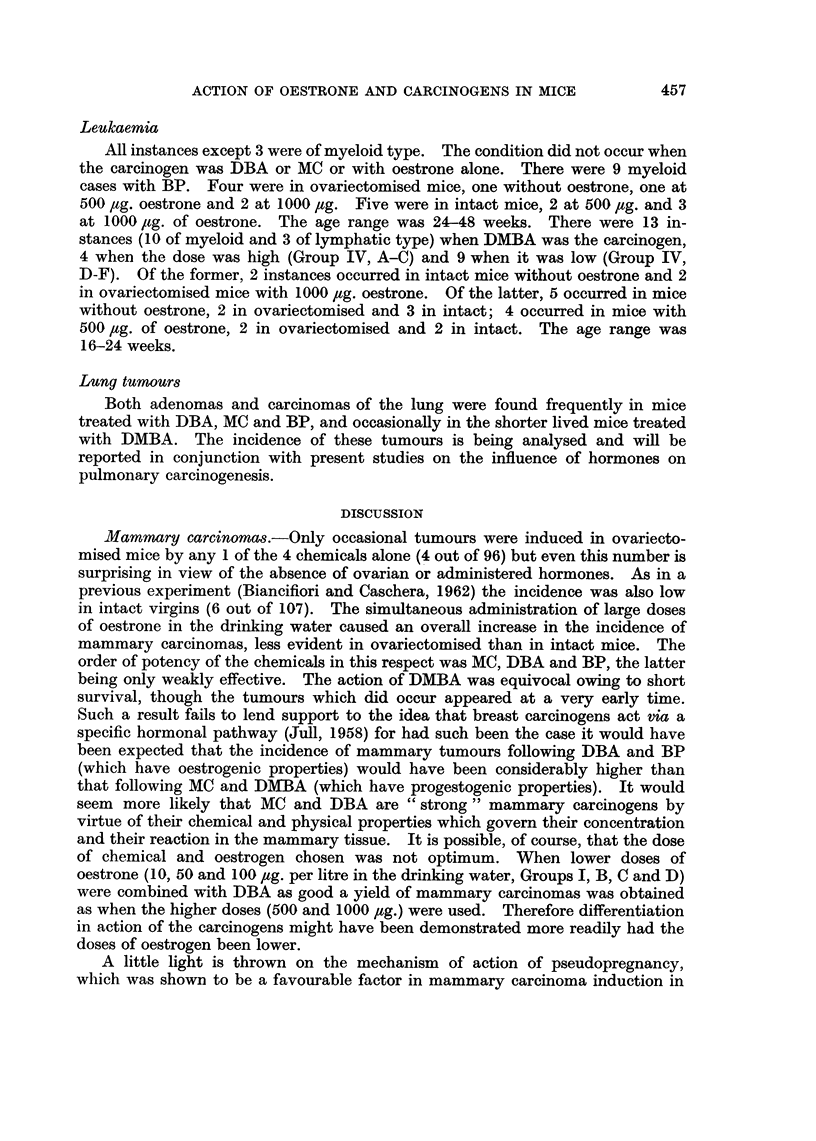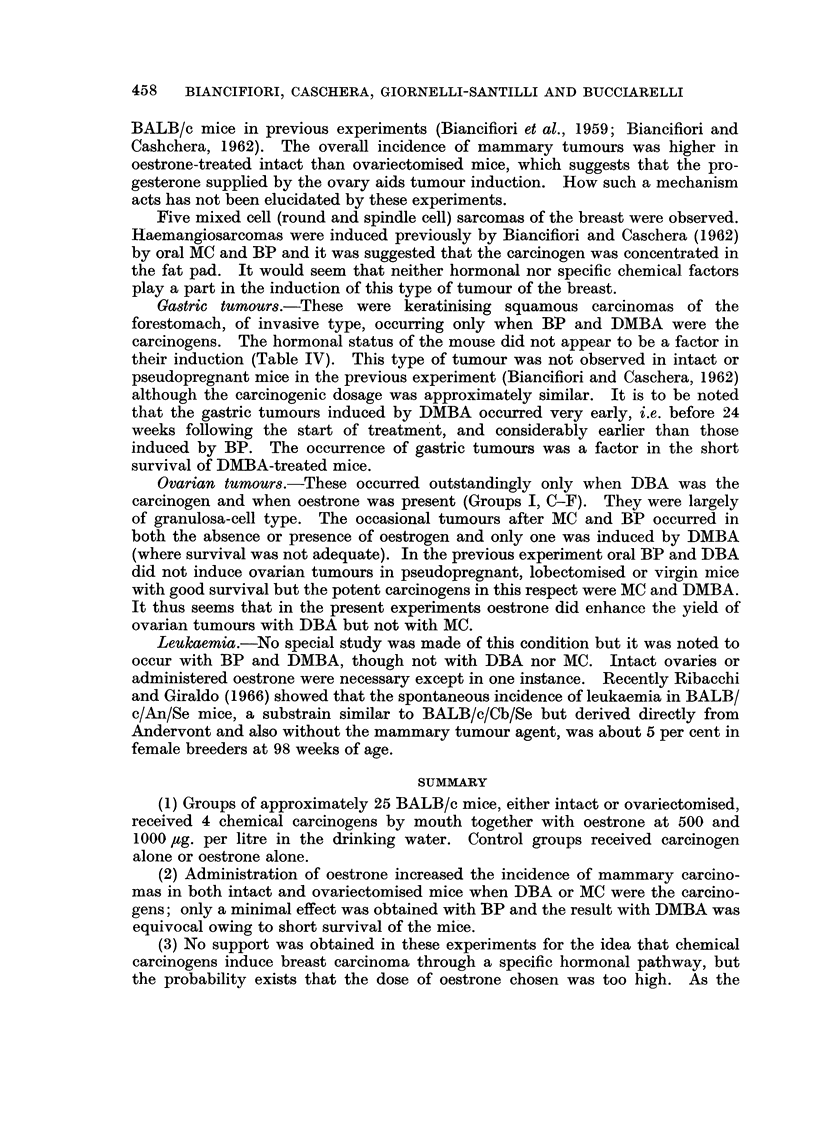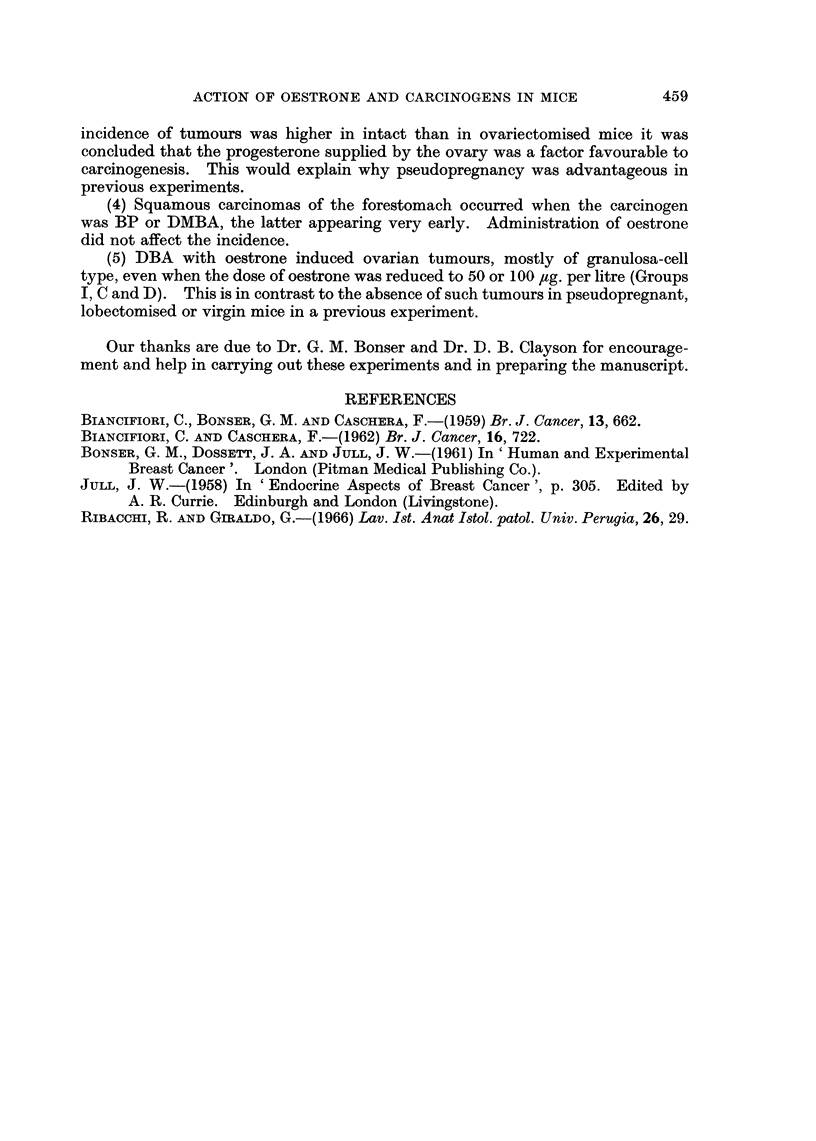# The Action of Oestrone and Four Chemical Carcinogens in Intact and Ovariectomised BALB/c/Cb/Se Mice

**Published:** 1967-06

**Authors:** C. Biancifiori, F. Caschera, F. E. Giornelli-Santilli, E. Bucciarelli


### THE ACTION OF OESTRONE AND FOUR CHEMICAL CARCINOGENS IN INTACT AND OVARIECTOMISED BALB/c/Cb/Se MICE

C. BIANCIFIORI, F. CASCHERA, F. E. GIORNELLI-SANTILLI AND E. BUCCIARELLI

*From the Division of Cancer Research, University of Study, Perugia, Italy*

Received for publication January 9, 1967

IN a previous experiment (Biancifiori, Bonser and Caschera, 1959) it was shown that a limited dose of 20-methylcholanthrene (MC) failed to induce mammary tumours in virgin or lobectomised BALB/c mice but that if the mice were made pseudopregnant 44 per cent developed mammary carcinomas. In a further experiment (Biancifiori and Cashera, 1962) it was shown that a larger dose of MC still caused only occasional tumours in lobectomised or virgin mice, but that 33 per cent of pseudopregnant mice developed tumours. It was also shown that pseudopregnancy enhanced the tumour yield over that in virgins when the carcinogen was 9,10-dimethyl-1,2-benzanthracene (DMBA), 1,2 : 5,6-dibenzanthracene (DBA) or 3,4-benzopyrene (BP). It was concluded that pseudopregnancy supplied two factors contributory to carcinogenesis by these chemicals, oestrogen and progesterone, but there was no information as to whether both hormones were necessary. The present experiments were undertaken with a dual purpose: firstly, to find out whether pseudopregnancy could be replaced by oestrogen alone if given continuously and secondly, to investigate whether the action of oestrogen would be similar when the carcinogen was varied. The idea of an oestrogen or progesterone pathway through which chemical carcinogens might exert their action had been postulated by Jull (1958).

### MATERIAL AND METHODS

Nine hundred and eight virgin female BALB/c/Cb/Se mice were used. Seven hundred and eighty survived for sufficient time to be reported, 403 intact and 377 ovariectomised (Table I).

Ovariectomy was performed through double flank incisions, under ether anaesthesia, at about 6 weeks of age. Thereafter both normal and castrated mice were maintained, 5 to a cage, on the standard pelleted diet of the laboratory.

Oestrone (The British Drug Houses, Ltd.) was dissolved in the drinking water at the required level and was presented to the mice *ad libitum* 1 week before chemical treatment was started. The oestrone was changed every second day, and was administered until the death of the animals.

DMBA, DBA, MC and BP (L. Light and Co. Ltd.) were dissolved in almond oil at a concentration of 0·5 per cent; 0·1 ml. (0·5 mg.) was administered by stomach tube twice weekly to the mice when they reached 8 weeks of age. This treatment was continued for 15 weeks. As DMBA at this level was too toxic, experiments IV D, IV E and IV F were started, in which the carcinogen was given for 8 weeks only.

TABLE I.—*Chemicals Administered to the Mice Used in These Experiments*

| Experiment | Chemical | Dose (mg.) | Oestrone (µg. per litre) | Number of effective mice* | |
|---|---|---|---|---|---|
| | | | | Intact | Ovariectomised |
| I G | — | — | 500 | 24(25) | 25(25) |
| I H | — | — | 1000 | 24(24) | 27(27) |
| I A | DBA | 15 | — | 20(22) | 19(21) |
| I B | DBA | 15 | 10 | 14(15) | 19(21) |
| I C | DBA | 15 | 50 | 25(25) | 10(16) |
| I D | DBA | 15 | 100 | 21(21) | 20(21) |
| I E | DBA | 15 | 500 | 19(19) | 19(21) |
| I F | DBA | 15 | 1000 | 20(25) | 17(17) |
| II A | MC | 15 | — | 25(25) | 25(25) |
| II B | MC | 15 | 500 | 25(25) | 24(24) |
| II C | MC | 15 | 1000 | 21(23) | 24(25) |
| III A | BP | 15 | — | 25(25) | 25(25) |
| III B | BP | 15 | 500 | 25(25) | 23(25) |
| III C | BP | 15 | 1000 | 26(26) | 20(21) |
| IV A | DMBA | 15 | — | 24(25) | 18(25) |
| IV B | DMBA | 15 | 500 | 17(25) | 19(25) |
| IV C | DMBA | 15 | 1000 | 17(25) | 15(25) |
| IV D | DMBA | 8 | — | 13(20) | 9(20) |
| IV E | DMBA | 8 | 500 | 11(20) | 7(20) |
| IV F | DMBA | 8 | 1000 | 7(20) | 12(20) |
| | | | | 403(459) | 377(449) |

* Mice which survived to the date of appearance of the first tumour caused by each chemical or until 51 weeks of age when no chemical was given. Figures in parenthesis represent the number of mice at the start.

The minimum time the mice had to survive to be included in the results of the experiments was determined by the interval between the first administration of the carcinogen and the discovery of the first tumour of any organ with that carcinogen. This period was: 4 and 7 weeks with 15 and 8 mg. DMBA respectively; 16 weeks with DBA; 24 weeks with BP and 36 weeks with MC. In experiments I G and I H, where no carcinogen was administered, the first effective mouse died aged 52 weeks, *i.e.* 44 weeks after the time at which the carcinogens had been administered to the other mice.

### RESULTS

### *Mammary carcinomas*

In Table II is stated the incidence of mammary carcinomas in the groups of intact and ovariectomised mice treated as shown in Table I. Table III is a summary of the chemical and the oestrogenic effects, the numbers of tumours occurring with 500 and 1000 µg./l. of oestrogen being considered together.

The general effect of a combination of oestrone and chemical was to increase the number of tumours above that induced by chemical or hormone alone, both in intact and ovariectomised mice (Table III). Usually the higher dose of 1000 µg. per litre in the drinking water was more effective than a lower dose of 500 µg. per litre (Table II). However, in the one experiment where lower doses of oestrone were tried (Expt. I, B–D) a considerable effect was obtained in intact mice at doses of 10, 50 and 100 µg. of oestrone per litre.

TABLE II.—*Mammary Carcinomas in Intact and Ovariectomised BALB/c Mice Treated with Chemicals and Oestrone*

| | | | Intact | | | | Ovariectomised | | | |
|---|---|---|---|---|---|---|---|---|---|---|
| | | Dose of oestrone | | Mammary carcinomas | | | | Mammary carcinomas | | |
| Exper. | Chemical | (μg. per litre) | Survival (weeks) | No. | Per cent | Mean age | Survival (weeks) | No. | Per cent | Mean age |
| I A | DBA | None | 16–44 | 1/20 | 5 | 26 | 22–44 | 0/19 | 0 | — |
| B | | 10 | 27–52 | 3/14 | 21 | 48 | 23–52 | 0/19 | 0 | — |
| C | | 50 | 27–52 | 10/25 | 40 | 39 | 27–54 | 1/10 | 10 | 38 |
| D | | 100 | 24–48 | 8/21 | 38 | 38 | 23–47 | 1/20 | 5 | 28 |
| E | | 500 | 25–38 | 3/19 | 16 | 35 | 23–41 | 1/19 | 5 | 25 |
| F | | 1000 | 16–46 | 11/20 | 55 | 25 | 24–26 | 3/17 | 18 | 31 |
| II A | MC | None | 41–54 | 4/25 | 16 | 53 | 43–61 | 2/25 | 8 | 56 |
| B | | 500 | 45–59 | 13/25 | 52 | 49 | 45–59 | 4/24 | 17 | 53 |
| C | | 1000 | 36–44 | 16/21 | 76 | 40 | 36–44 | 7/24 | 28 | 41 |
| III A | BP | None | 28–65 | 2/25 | 8 | 57 | 24–64 | 0/25 | 0 | — |
| B | | 500 | 26–60 | 4/25 | 16 | 39 | 24–58 | 0/23 | 0 | — |
| C | | 1000 | 26–56 | 2/26 | 8 | 37 | 28–54 | 1/20 | 5 | 48 |
| IV A | DMBA | None | 4–14 | 0/24 | 0 | — | 7–15 | 2/18 | 11 | 10 |
| B | (15) | 500 | 7–14 | 1/17 | 6 | 7 | 7–11 | 0/19 | 0 | — |
| C | | 1000 | 8–15 | 2/17 | 12 | 9 | 8–15 | 2/15 | 13 | 10 |
| IV D | DMBA | None | 8–21 | 0/13 | 0 | — | 7–21 | 0/9 | 0 | — |
| E | (8) | 500 | 9–23 | 4/11 | 36 | 13 | 7–24 | 0/7 | 0 | — |
| F | | 1000 | 9–20 | 1/7 | 14 | 17 | 7–21 | 2/12 | 17 | 12 |
| I G | None | 500 | 61–65 | 0/24 | 0 | — | 45–64 | 0/25 | 0 | — |
| H | | 1000 | 51–63 | 0/24 | 0 | — | 44–63 | 0/27 | 0 | — |

Figures in parenthesis represent total oral dose (mg.) of DMBA.

TABLE III.—*Summary of Effect of Oestrone on Mammary Tumour Induction*

| | | | Intact Mammary tumours | | Ovariectomised Mammary tumours | |
|---|---|---|---|---|---|---|
| Exper. | Chemical | Presence or absence of oestrone | No. | Per cent | No. | Per cent |
| I | DBA | None | 1/20 | 5 | 0/19 | 0 |
| | | 500 or 1000 μg. | 14/39 | 36 | 4/36 | 11 |
| II | MC | None | 4/25 | 16 | 2/25 | 8 |
| | | 500 or 1000 μg. | 29/46 | 63 | 11/48 | 23 |
| III | BP | None | 2/25 | 8 | 0/25 | 0 |
| | | 500 or 1000 μg. | 6/51 | 12 | 1/43 | 2 |
| IV | DMBA | None | 0/24 | 0 | 2/18 | 11 |
| | (15) | 500 or 1000 μg. | 3/34 | 9 | 2/34 | 6 |
| | DMBA | None | 0/13 | 0 | 0/9 | 0 |
| | (8) | 500 or 1000 μg. | 5/18 | 28 | 2/19 | 11 |
| I | None | 500 or 1000 μg. | 0/48 | 0 | 0/52 | 0 |

Figures in parenthesis represent total oral dose of (mg.) DMBA.

It is now necessary to analyse the results obtained in individual groups of mice.

*Ovariectomised mice* (Table II).—The effect of oestrone at both the doses given (natural progesterone being absent) can be seen. Mammary carcinoma induction by DBA and MC was appreciably increased, but that by BP was not increased.

Any effect by DMBA was equivocal, owing to the short survival of the mice, especially of those which had the high dose of DMBA (Groups IV, A–C). But the 14 tumours which did occur appeared at mean ages ranging from 7–17 weeks, which can be regarded as early. Oestrone alone failed to induce mammary carcinomas in spite of long survival (Groups I, G and H).

*Intact mice.*—The effect of oestrone (progesterone being supplied by the ovary) was rather greater than in ovariectomised mice. DBA and MC induced the highest number of tumours (Table III), the effect of BP was minimal and that with DMBA equivocal, owing to the early death of the mice.

All the tumours in ovariectomised and intact mice were confirmed microscopically as mammary adenocarcinomas, of the type associated with chemical induction (Bonser, Dossett and Jull, 1962). One tumour was classified as a carcinosarcoma and in 3, areas of squamous metaplasia were observed. In addition there were 5 mammary sarcomas, which are not included in the analysis of carcinomas. Three were in mice treated with DBA. Two of these mice had received no oestrone; one was intact and was killed at 16 weeks, while the other was an ovariectomised mouse and died at 29 weeks. The third DBA sarcoma occurred in an ovariectomised mouse which had received a high level of oestrone and was killed at 30 weeks. The other 2 sarcomas arose in mice treated with MC. One was ovariectomised and received 1000 $\mu$g./l. oestrone until killed at 37 weeks; the other was intact, receiving 500 $\mu$g./l. oestrone until killed at 51 weeks.

*Gastric tumours*

None occurred with DBA or MC but there were many following treatment with BP and DMBA, whether oestrone was administered or not and whether the mice were ovariectomised or intact (Table IV). The more effective carcinogen was DMBA, the first tumour occurring at 4 weeks and all the tumours having occurred by 22 weeks. With BP the range of tumour induction was 26–60 weeks.

The gastric tumours were all squamous carcinomas of the forestomach, with varying degrees of infiltration of the sub-epithelial tissue and muscular wall. One gastric carcinoma induced by BP and found at 29 weeks had invaded both ovaries by spreading across the peritoneum.

*Ovarian tumours*

The highest incidence was obtained in mice treated with DBA and oestrone. None occurred following treatment with DBA alone or oestrogen alone (Groups I, A, G and H) nor with the lowest dose of oestrone (Group I, B). Eight tumours occurred when the dose of oestrone was 50 or 100 $\mu$g. per litre (Groups I, C and D) and 9 when the higher doses were given (Groups I, E and F), the numbers of effective mice being 46 and 39 respectively (Table I). The latent period was 17–39 weeks. Twelve of the tumours were classified as of granulosa-cell origin, 4 as luteomas and 1 as mixed.

Three ovarian tumours occurred in group II A and 2 in group III A, where MC or BP was administered without oestrogen. The mean latent period with MC was 50 weeks and with BP 62 weeks. One tumour occurred at 51 weeks with MC and 500 $\mu$g. oestrone (Group II, B), one at 48 weeks with BP and 1000 $\mu$g. (Group III, C) and another at 20 weeks with DMBA and 1000 $\mu$g. oestrone (Group IV, F). All these 8 tumours were of granulosa-cell type.

19

TABLE IV.—*Incidence and Mean Age of Gastric Tumours*

| | | | Ovariectomised | | | | | Intact | | | | |
|---|---|---|---|---|---|---|---|---|---|---|---|---|
| | | | | | Gastric tumours | | | | | Gastric tumours | | |
| Exper. | Chemicals | Dose of oestrone (μg.) | No. of effective mice | Survival following start of treatment (weeks) | No. | Per cent | Mean age | No. of effective mice | Survival following start of treatment (weeks) | No. | Per cent | Mean age |
| III A | BP | None | 25 | 24–64 | 4 | 16 | 44 | 25 | 28–65 | 5 | 20 | 43 |
| B | | 500 | 23 | 24–58 | 2 | 8 | 36 | 25 | 26–60 | 4 | 16 | 41 |
| C | | 1000 | 20 | 28–54 | 2 | 10 | 47 | 26 | 26–56 | 6 | 23 | 38 |
| IV A | DMBA (15) | None | 18 | 7–15 | 4 | 22 | 14 | 24 | 4–14 | 10 | 42 | 9 |
| B | | 500 | 19 | 7–11 | 4 | 21 | 8 | 17 | 7–14 | 8 | 47 | 9 |
| C | | 1000 | 15 | 8–15 | 3 | 20 | 11 | 17 | 8–15 | 9 | 53 | 10 |
| IV D | DMBA (8) | None | 9 | 7–21 | 5 | 56 | 12 | 13 | 8–21 | 6 | 46 | 9 |
| E | | 500 | 7 | 7–24 | 2 | 29 | 8 | 11 | 9–23 | 3 | 27 | 15 |
| F | | 1000 | 12 | 7–21 | 5 | 42 | 12 | 7 | 9–20 | 2 | 29 | 13 |

*Leukaemia*

All instances except 3 were of myeloid type. The condition did not occur when the carcinogen was DBA or MC or with oestrone alone. There were 9 myeloid cases with BP. Four were in ovariectomised mice, one without oestrone, one at 500 $\mu$g. oestrone and 2 at 1000 $\mu$g. Five were in intact mice, 2 at 500 $\mu$g. and 3 at 1000 $\mu$g. of oestrone. The age range was 24–48 weeks. There were 13 instances (10 of myeloid and 3 of lymphatic type) when DMBA was the carcinogen, 4 when the dose was high (Group IV, A–C) and 9 when it was low (Group IV, D-F). Of the former, 2 instances occurred in intact mice without oestrone and 2 in ovariectomised mice with 1000 $\mu$g. oestrone. Of the latter, 5 occurred in mice without oestrone, 2 in ovariectomised and 3 in intact; 4 occurred in mice with 500 $\mu$g. of oestrone, 2 in ovariectomised and 2 in intact. The age range was 16–24 weeks.

*Lung tumours*

Both adenomas and carcinomas of the lung were found frequently in mice treated with DBA, MC and BP, and occasionally in the shorter lived mice treated with DMBA. The incidence of these tumours is being analysed and will be reported in conjunction with present studies on the influence of hormones on pulmonary carcinogenesis.

### DISCUSSION

*Mammary carcinomas.*—Only occasional tumours were induced in ovariectomised mice by any 1 of the 4 chemicals alone (4 out of 96) but even this number is surprising in view of the absence of ovarian or administered hormones. As in a previous experiment (Biancifiori and Caschera, 1962) the incidence was also low in intact virgins (6 out of 107). The simultaneous administration of large doses of oestrone in the drinking water caused an overall increase in the incidence of mammary carcinomas, less evident in ovariectomised than in intact mice. The order of potency of the chemicals in this respect was MC, DBA and BP, the latter being only weakly effective. The action of DMBA was equivocal owing to short survival, though the tumours which did occur appeared at a very early time. Such a result fails to lend support to the idea that breast carcinogens act *via* a specific hormonal pathway (Jull, 1958) for had such been the case it would have been expected that the incidence of mammary tumours following DBA and BP (which have oestrogenic properties) would have been considerably higher than that following MC and DMBA (which have progestogenic properties). It would seem more likely that MC and DBA are " strong " mammary carcinogens by virtue of their chemical and physical properties which govern their concentration and their reaction in the mammary tissue. It is possible, of course, that the dose of chemical and oestrogen chosen was not optimum. When lower doses of oestrone (10, 50 and 100 $\mu$g. per litre in the drinking water, Groups I, B, C and D) were combined with DBA as good a yield of mammary carcinomas was obtained as when the higher doses (500 and 1000 $\mu$g.) were used. Therefore differentiation in action of the carcinogens might have been demonstrated more readily had the doses of oestrogen been lower.

A little light is thrown on the mechanism of action of pseudopregnancy, which was shown to be a favourable factor in mammary carcinoma induction in

BALB/c mice in previous experiments (Biancifiori *et al.*, 1959; Biancifiori and Cashchera, 1962). The overall incidence of mammary tumours was higher in oestrone-treated intact than ovariectomised mice, which suggests that the progesterone supplied by the ovary aids tumour induction. How such a mechanism acts has not been elucidated by these experiments.

Five mixed cell (round and spindle cell) sarcomas of the breast were observed. Haemangiosarcomas were induced previously by Biancifiori and Caschera (1962) by oral MC and BP and it was suggested that the carcinogen was concentrated in the fat pad. It would seem that neither hormonal nor specific chemical factors play a part in the induction of this type of tumour of the breast.

*Gastric tumours.*—These were keratinising squamous carcinomas of the forestomach, of invasive type, occurring only when BP and DMBA were the carcinogens. The hormonal status of the mouse did not appear to be a factor in their induction (Table IV). This type of tumour was not observed in intact or pseudopregnant mice in the previous experiment (Biancifiori and Caschera, 1962) although the carcinogenic dosage was approximately similar. It is to be noted that the gastric tumours induced by DMBA occurred very early, *i.e.* before 24 weeks following the start of treatment, and considerably earlier than those induced by BP. The occurrence of gastric tumours was a factor in the short survival of DMBA-treated mice.

*Ovarian tumours.*—These occurred outstandingly only when DBA was the carcinogen and when oestrone was present (Groups I, C–F). They were largely of granulosa-cell type. The occasional tumours after MC and BP occurred in both the absence or presence of oestrogen and only one was induced by DMBA (where survival was not adequate). In the previous experiment oral BP and DBA did not induce ovarian tumours in pseudopregnant, lobectomised or virgin mice with good survival but the potent carcinogens in this respect were MC and DMBA. It thus seems that in the present experiments oestrone did enhance the yield of ovarian tumours with DBA but not with MC.

*Leukaemia.*—No special study was made of this condition but it was noted to occur with BP and DMBA, though not with DBA nor MC. Intact ovaries or administered oestrone were necessary except in one instance. Recently Ribacchi and Giraldo (1966) showed that the spontaneous incidence of leukaemia in BALB/c/An/Se mice, a substrain similar to BALB/c/Cb/Se but derived directly from Andervont and also without the mammary tumour agent, was about 5 per cent in female breeders at 98 weeks of age.

### SUMMARY

(1) Groups of approximately 25 BALB/c mice, either intact or ovariectomised, received 4 chemical carcinogens by mouth together with oestrone at 500 and 1000 $\mu$g. per litre in the drinking water. Control groups received carcinogen alone or oestrone alone.

(2) Administration of oestrone increased the incidence of mammary carcinomas in both intact and ovariectomised mice when DBA or MC were the carcinogens; only a minimal effect was obtained with BP and the result with DMBA was equivocal owing to short survival of the mice.

(3) No support was obtained in these experiments for the idea that chemical carcinogens induce breast carcinoma through a specific hormonal pathway, but the probability exists that the dose of oestrone chosen was too high. As the incidence of tumours was higher in intact than in ovariectomised mice it was concluded that the progesterone supplied by the ovary was a factor favourable to carcinogenesis. This would explain why pseudopregnancy was advantageous in previous experiments.

(4) Squamous carcinomas of the forestomach occurred when the carcinogen was BP or DMBA, the latter appearing very early. Administration of oestrone did not affect the incidence.

(5) DBA with oestrone induced ovarian tumours, mostly of granulosa-cell type, even when the dose of oestrone was reduced to 50 or 100 μg. per litre (Groups I, C and D). This is in contrast to the absence of such tumours in pseudopregnant, lobectomised or virgin mice in a previous experiment.

Our thanks are due to Dr. G. M. Bonser and Dr. D. B. Clayson for encouragement and help in carrying out these experiments and in preparing the manuscript.

### REFERENCES

Biancifiori, C., Bonser, G. M. and Caschera, F.—(1959) *Br. J. Cancer*, **13**, 662.

Biancifiori, C. and Caschera, F.—(1962) *Br. J. Cancer*, **16**, 722.

Bonser, G. M., Dossett, J. A. and Jull, J. W.—(1961) In 'Human and Experimental Breast Cancer'. London (Pitman Medical Publishing Co.).

Jull, J. W.—(1958) In 'Endocrine Aspects of Breast Cancer', p. 305. Edited by A. R. Currie. Edinburgh and London (Livingstone).

Ribacchi, R. and Giraldo, G.—(1966) *Lav. Ist. Anat Istol. patol. Univ. Perugia*, **26**, 29.